# Health Care Providers’ Perspective and Knowledge about Peri-Surgical Medication and Practices in Breastfeeding Women

**DOI:** 10.3390/ijerph20043379

**Published:** 2023-02-15

**Authors:** Lena De Hondt, Santina Lisa Gorsen, Patrick Verburgh, Kristien De Paepe, Joke Muyldermans, Eline Tommelein

**Affiliations:** 1 Faculty of Medicine and Pharmacy, Department of Pharmaceutical and Pharmacological Sciences, Vrije Universiteit Brussel, Laerbeeklaan 103, 1090 Jette, Belgium; 2 Faculty of Medicine and Pharmacy, Department of Pharmaceutical and Pharmacological Sciences, In Vitro Toxicology and Dermato-Cosmetology, Vrije Universiteit Brussel, Laerbeeklaan 103, 1090 Jette, Belgium; 3 Ziekenhuis Netwerk Antwerpen Stuivenberg, Langebeeldekensstraat 267, 2060 Antwerpen, Belgium

**Keywords:** analgesics, general anesthetics, breastfeeding, health care professionals, lactating, local anesthetics, surgical procedures

## Abstract

Many guidelines offer recommendations to support the continuation of breastfeeding and the choice of medication when a mother undergoes a surgical procedure. The aim of this study is to investigate health care providers’ (HCPs) current practices and knowledge about peri-surgical medication and practices in breastfeeding women. We performed a cross-sectional study in Flanders (Belgium) assessing demographics, beliefs about breastfeeding and its health benefits, current practices concerning breastfeeding women undergoing (surgical) procedures and specific knowledge about the use of medication during breastfeeding. Two hundred and ninety-one (291) participants completed the online questionnaire. Many participants considered their knowledge about breastfeeding to be good, and almost all participants acknowledged the superiority of breastfeeding and the importance of its continuation. Very few participants were, however, familiar with the available protocols concerning surgical procedures in breastfeeding women. Less than half of the participants routinely advised the recommended practices to protect breastfeeding. For most of the peri-surgical medication, participants needed to look-up information about the compatibility with breastfeeding. We conclude that there is a knowledge gap and recommend the development of a comprehensive guideline as well as implementation of this information in basic and post-academic training.

## 1. Introduction

When a lactating mother undergoes a surgical procedure with (general) anesthesia, the success of breastfeeding continuation is influenced by the choice of medication and additional measures before, during and after the procedure [1]. However, there is little rigorous information on the application of these measures, nor on the rational use of anesthesia-related medication during the breastfeeding period. Current recommendations are mostly defined based on the pharmacodynamics and pharmacokinetics of medication, limited studies of milk concentrations and rare effects on infants [2]. Nevertheless, multiple published guidelines conclude that most mothers can safely breastfeed following anesthesia when they are awake and alert [2,3,4,5,6].

Historically, mothers were often informed to “pump and dump” their breast milk for 24 to 48 h after receiving anesthesia to avoid passing medication to the infant [7,8]. Although this advice might seem the safest to protect the infant from any medication intake through the breastmilk, it is harmful when unnecessary. A great amount of the medication used during surgical procedures does not transfer to breastmilk, or is eliminated over a short period of time [9]. An unnecessary and prolonged interruption of breastfeeding can subsequently negatively impact/both mother and child as it increases the risks of mastitis and the decline of milk production [7]. The latter two can lead to the unwanted early cessation of breastfeeding, which negatively affects the health of both mother and child [2,10].

Besides correct advice concerning medication, a number of peri-surgical practices can be implemented to protect the breastfeeding process [1,9,11,12]. These include the recommendation to consider regional or epidural anesthesia when possible. Second, it should be possible to bring the nursing child to the hospital together with a responsible adult to care for the infant during the intended procedure [1]. If this is not possible, pumping equipment should be available. The mother must have the possibility to breastfeed or express as much milk as possible before the procedure to help prevent engorgement. Expressed breastmilk should be adequately stored and given to the infant during or after the procedure when the mother is not (yet) able to breastfeed. Finally, the mother should be able to breastfeed or pump as soon as she is alert and awake.

The aim of the current study is to map the knowledge and practices of peri-surgical medication and measures for breastfeeding women from the health care providers’ (HCPs) perspective. Additionally, we questioned HCPs about their attitude towards breastfeeding and the specific knowledge about the use of mainly anesthetics, analgesics and adjuvants in breastfeeding mothers during anesthesia or procedural sedation.

## 2. Materials and Methods

### 2.1. Design

A cross-sectional study was performed researching the current practices when women had to undergo a (surgical) procedure during the breastfeeding period, regardless of how long it had been after delivery. The study was carried out in Flanders (Dutch-speaking part of Belgium) from January 2021 until April 2021. An approval was obtained by the ethical committee of the University Hospital of Brussels (number: 1432021000389; principal investigator: Eline Tommelein; date of approval 3 February 2021). Every participant provided written consent before commencing the questionnaire.

This manuscript adheres to the applicable STROBE guidelines.

### 2.2. Participants and Data Collection

Different HCPs could participate in the study: community pharmacists, medical specialists (anesthesiologists, gynecologists, obstetricians, orthopedists, emergency room physicians, intensive care unit physicians and pediatricians), general practitioners, lactation consultants, but also dentists, (peri-operative) nurses and midwives, the latter of whom are authorized to prescribe medication within their area of expertise, and, in Belgium, are generally consulted by women up until 1 year after delivery. As well, midwives are the main point of contact for breastfeeding-related questions, even after the one-year postpartum date. A specialization as a lactation consultant was not required for participation as currently, in Belgium, the title of lactation consultant is not recognized by the government. Nevertheless, a postgraduate college program or the certification of the International Board-Certified Lactation Consultants (IBCLC) is perceived as a sufficient qualification. Training on lactation for all staff is however required when a hospital wants to obtain a BFHI certificate.

All HCPs were contacted through their respective professional organization, newsletters, websites, e-mails and social media channels. Questionnaires were answered online.

### 2.3. Outcomes

First, the study collected the basic demographic data of the participants (Table 1). Second, common beliefs about breastfeeding and its health benefits were assessed (Table 2), followed by an evaluation of current practices concerning breastfeeding women undergoing (surgical) procedures (Table 3 and Table 4). Finally, knowledge about the compatibility of medication used in surgical procedures in breastfeeding mothers was assessed (Table 3 and on-line Supplementary Material). The included drug groups were analgesics, adjuvants, anticoagulants and antiaggregants, antibiotics, (local) anesthetics and antiemetics.

Surgical procedures were defined as any procedure where an incision through tissue is made. Examples include the removal of a mole or a torn toenail and dental procedures, but also procedures with epidural or general anesthesia. Procedures could be related to delivery but could also take place months after in the context of a different condition. Participants were asked to reflect on all procedures they came into contact with. For example, gynecologists could reflect on experiences related to pregnancy (i.e., caesarean section), but also gynecological procedures in general for women who happen to be breastfeeding, such as in the case of the removal of a breast abscess.

### 2.4. Statistical Analysis

Data were analyzed using Microsoft^®^ Excel^®^ and SPSS^®^. Results are presented descriptively with means and standard deviations and median and interquartile ranges as appropriate. The statistical analysis plan was approved by the authors before analyses began.

## 3. Results

### 3.1. Demographic and Basic Data

After dissemination of the online questionnaire, 291 HCPs participated in the study. The largest group included 92 midwives or 31.6% of the respondents. Eighty-nine (89) medical specialists (30.6%) formed the second largest group which consisted of fifty-six anesthesiologists, twenty-seven gynecologists/obstetricians, one pediatrician, one orthopedist, two intensive care physicians and two emergency care physicians. In all HCP groups, it can be noted that for most participants—when having children of their own—these children were exclusively breastfed, even if the breastfeeding period only was a few weeks. Of all 291 participants, 114 (39.2%), 31 (45.0%) and 18 participants (6.2%) believed their own knowledge about breastfeeding was mediocre, good or very good, respectively. A detailed overview of demographic and basic data are provided in Table 1.

### 3.2. Beliefs about Breastfeeding and Its Health Benefits 

The attitude of the respondents towards the WHO guidelines about the duration of (exclusive) breastfeeding was rather mixed with 23 (7.9%), 71 (24.4%), 111 (38.1%) and 86 participants (29.6%) having a respective negative, neutral, positive or very positive attitude. Almost all participants (276; 94.8%), however, acknowledged the superiority of breastfeeding over infant formula for a child’s nutrition. In total, 132 (54.4%) and 135 participants (46.4%) considered the continuation of breastfeeding after a surgical procedure as important or very important (Table 2).

### 3.3. Knowledge of Guidelines and Advice Concerning Peri-Surgical Measures in Breastfeeding Women

Overall, about 75% of participants stated they did not know about simple and clear protocols considering breastfeeding women undergoing a (surgical) procedure in general. Additionally, only 54 participants (19.8%) indicated that there was a protocol available at their workspace. The participating HCPs were only very limitedly acquainted with the published guidelines, and it is to be noted that none of the participants stated to be familiar with the guidelines from the Academy of Breastfeeding Medicine or the guidelines from The Breastfeeding Network (Table 3) [1,31].

During routine practice, more than half of the participants never or very rarely inquired about breastfeeding practices with women of a fertile age (Table 4). Ten percent of the participants stated to always give the same advice, such as “pump and dump for 24 h”.

Considering peri-surgical measures, the most provided advice towards women included: “Bringing a responsible caretaker for the nursing child”, “Expressing milk before the procedure and store for the day of the procedure” and “Breastfeeding or expressing milk right before the procedure”. None of the other measures in the published protocols were substantially often mentioned. An extra question specifically for the medical specialists and peri-operative nurses was added regarding to what extent they considered using locoregional instead of general anesthesia for women who are breastfeeding. Of the medical specialists, 68 respondents (76%) took this into consideration. Of the peri-operative nurses, it concerned six participants (33%).

### 3.4. Knowledge of Compatibility of Medication Used during (Surgical) Procedures in Breastfeeding Women

Only 38 participants (13.1%) stated that there was sufficient attention paid to the subject of medication use during breastfeeding throughout their academic education. No less than 260 respondents (89.3%) were interested in learning more about this topic. The most consulted sources to evaluate the safety of a drug during breastfeeding are Cybele [Belgian database] (138; 47.4%). Lareb [Dutch database] (95; 32.6%), the Summary of Product Characteristics (79; 21.1%) and Lactmed [United States database] (73; 25.1%) (Table 3). When insufficient data are available, HCPs make use of pharmacokinetic and other parameters to make an estimation of its safety. However, the number of considered parameters remains limited. HCPs seem to consult their colleagues on this topic. More details can be found in Table S1 (online Supplementary Materials).

In general, HCPs rarely advise the cessation of breastfeeding when a specific drug is contra-indicated (online Supplementary Materials). When a contra-indication is present, the included HCPs preferred to work with a different molecule. As well, a noteworthy number of participants regularly stated to look-up information on a particular compatibility when not knowing it by heart. 

Considering the *analgesics*, the compatibility of frequently prescribed or dispensed drugs such as paracetamol, ibuprofen and diclofenac is known. Opioids, on the other hand, are often incorrectly considered incompatible; however, still one-fourth of the participants looked this information up per molecule (online Supplementary Materials Table S2). Considering the *adjuvants*, responses throughout the HCPs were very diverse (online Supplementary Material Table S3). The knowledge about the compatibility of *anticoagulants* and *antiaggregants* was very good. Almost all HCPs that work with the questioned medication knew that they are compatible (online Supplementary Materials Table S4). For the regularly used *antibiotics*, amoxicillin (with clavulanic acid) compatibility was known by most HCPs. For the other *antibiotics*, compatibility was recognized when they worked with the drug (online Supplementary Materials Table S5). Compatibility of (local) *anesthetics* such as lidocaine was well known by HCPs that work with this medicine. When not known by heart, the information was looked up (online Supplementary Materials Table S6). Finally, *antiemetics* were largely considered compatible, even though all guidelines consider the risks associated with the use of alizapride, ondansetron and droperidol ”possible” or “unknown” (online Supplementary Materials Table S7).

## 4. Discussion

A total of 291 HCPs were included in this cross-sectional study on the perspective and knowledge about peri-surgical medication and practices in breastfeeding women. First, they reported on their attitude towards breastfeeding. Subsequently, we mapped their practices concerning (surgical) procedures in breastfeeding women and their specific knowledge about the use of mainly anesthetics, analgesics and adjuvants in lactating women during anesthesia or procedural sedation. Many participants believe their knowledge about breastfeeding to be good, and almost all participants acknowledge the superiority of breastfeeding and the importance of its continuation after surgery. It was observed that very few participants are familiar with the existing protocols concerning this topic. Additionally, very few women of the fertile age appear to be questioned about whether they are breastfeeding. Less than half of the participants advise the recommended practices to protect breastfeeding in lactating women undergoing a surgical procedure. Knowledge about the compatibility of regularly used medications such as amoxicillin, paracetamol and ibuprofen with breastfeeding is good. For the majority of all other medications, HCPs need to look-up information due to not being familiar with the available and consultable sources.

Although a fair number of protocols concerning (surgical) procedures in breastfeeding women are available, to the best of our knowledge, only very limited practice research on this topic has been performed. This is the first study researching real-life practices concerning (surgical) procedures in breastfeeding women.

Many participants (45.0%) believe their knowledge about breastfeeding to be good; however, most dentists believe theirs is poor (27.8%) or mediocre (55.6%). Almost all participants in all groups acknowledge the superiority of breastfeeding and the importance of its continuation after surgery. This is a positive finding, as it was previously shown that physicians and nurses have an important influence on the initiation and duration of lactation [32]. DiGirolamo and others observed that the neutrality of HCPs concerning breastfeeding may negatively affect breastfeeding initiation and duration [33]. An interview study with breastfeeding mothers also showed that breastfeeding women believe that providing evidence-based breastfeeding support in a sensitive and individualized manner is valued very highly. This could be strengthened by encouragement and a positive approach by HCPs [34]. In Belgium, midwives play a key role in counselling breastfeeding. Furber and Thomson conducted a study exploring the breastfeeding-supporting role of midwives and their experiences in two maternity hospitals in the north of England between Autumn 1999 and Autumn 2001. One of the findings of this study was that the participants gathered together every day to reduce conflicting advice because contradictions could disempower mothers to breastfeed or weaken their self-confidence and ability to breastfeed [35]. In addition, Rosin and Grković emphasized the importance of mutual collaboration between practitioners and midwives in breastfeeding support [36].

It is remarkable that very few participants are familiar with the existing protocols concerning (surgical) procedures in breastfeeding women. Less than half of the respondents routinely advise the recommended practices to protect breastfeeding in lactating women undergoing a surgical procedure. This might be because there is no knowledge about them. Interestingly, even HCPs who work in a hospital with a BFHI certification indicated that their hospital does not provide a clear protocol. Nevertheless, some measures, such as planning a breastfeeding patient as the first of the day, although barely advised, can significantly improve breastfeeding outcomes.

In our study, less than 15% of participants state there is sufficient attention paid to the subject of medication use during breastfeeding throughout their academic education, and about 90% are interested in learning more about this topic. Indeed, McCarthy and others showed that this topic is indeed rarely present in curricula [37]. The current study also shows that the participants do not distinguish between information that can be consulted in primary sources such as Lactmed and secondary sources such as Lareb, while primary sources are more reliable [22,23]. Although the included HCPs perceive their knowledge about breastfeeding as “good”, the amount of medication that is incorrectly classified, or of which no knowledge is available, is substantial. Mccarthy et al. obtained similar results [37].

The current study shows that (repeated) education of all HCPs about breastfeeding, surgical procedures during breastfeeding and medication use during breastfeeding is adamant. First, it would be helpful for a comprehensive guideline including a summary of all available data specifically targeting the involved HCPs to be developed. Subsequently, to address the observed knowledge gap, we recommend an (online) learning module teaching how to retrieve best practice evidence considering all the aforementioned topics and apply the guideline. It has been shown that this type of educational intervention is able to improve this knowledge [11] This way, implementation in both basic and post-academic training of all HCPs will be facilitated. Finally, care facilities must provide clear protocols, in particular the hospitals with a BFHI certificate, and should be evaluated on the matter by the responsible governmental body. To further improve the current practice, adding certified lactation specialists to the standard health care team is recommended. For future research, it would be interesting to repeat this study on different time points in different countries and assess mothers’ opinions.

The significance of the data may be variable because we could not define the response rate as the questionnaires were sent out through professional organizations, newsletters, websites, e-mails and social media channels. Probably only HCPs with a specific interest in breastfeeding participated. However, the rate of their own exclusively breastfed children (even for a few weeks) is comparable with the Flemish mean numbers. According to the registration numbers of Kind en Gezin (agency of the Flemish government), 81.4% of newborns in Flanders receive breastmilk exclusively in the first day after birth. The following six days, this number decreases to 77% [38]. To additionally address this bias, questionnaires were sent to as much different professional associations as possible and were completed anonymously. This could also prevent socially desirable answers. Furthermore, we only obtained, respectively, 20, 18 and 18 respondents for the groups of the dentists, general practitioners and peri-operative nurses. This led to less reliable results, and differences were noticeable. However, the percentage of participants that indicated that they worked in a hospital with a BFHI certificate corresponds to the number of hospitals in Flanders that have a BFHI certificate, which could indicate that we collected participants equally around Flanders [39].

## 5. Conclusions

It can be concluded that there is a knowledge gap considering peri-surgical medications and practices in breastfeeding women. The study also revealed that HCPs are not familiar with breastfeeding-related guidelines in general, nor are they familiar with sources providing evidence-based information about medication use during breastfeeding. The development of a comprehensive guideline and including this information in basic and post-academic training are recommended.

## Figures and Tables

**Table 1:** Demographic and basic data of health care providers.

	Medical Specialists (*n* = 89)	Pharmacists (*n* = 54)	General Practitioners (*n* = 20)	Dentists (*n* = 18)	Peri-Operative Nurses (*n* = 18)	Midwives (*n* = 92)	All (*n* = 291)
**Age, mean ± SD (range)**	41.5 ± 11.2	36.1 ± 10.2	43.3 ± 14.2	47.2 ± 14.1	45.7 ± 10.9	37.3 ± 10.3	39.9 ± 11.5
**Female gender, *n* (%)**	51 (57.3%)	47 (87.0%)	16 (80.0%)	10 (55.6%)	17 (94.4%)	91 (98.9%)	232 (79.7%)
**Having children, *n* (%)**	69 (77.5%)	38 (70.4%)	15 (75.0%)	13 (72.2%)	16 (88.9%)	68 (73. 9%)	219 (75.3%)
Feeding choice for own children, *n* (%) *							
Exclusively FF	11 (15.9%)	8 (21.1%)	2 (13.3%)	4 (30.8%)	4 (25.0%)	21 (30.9%)	50 (22.8%)
Exclusively BF for at least a few weeks	52 (75.4%)	28 (73.7%)	11 (73.3%)	7 (53.8%)	12 (75.0%)	44 (64.7%)	154 (70.3%)
One/multiple child(ren) exclusively BF and one/multiple child(ren) exclusively FF	6 (8.7%)	2 (5.3%)	2 (13.3%)	2 (15.4%)	0 (0.0%)	3 (4.4%)	15 (6.8%)
**Training and education, *n* (%)**							
In training	21 (23.6%)	NA	4 (20.0%)	NA	NA	NA	25 (8.6%)
Additional degree as LC or IBCLC	2 (2.2%)	0 (0.0%)	0 (0.0%)	1 (5.6%)	3 (16.7%)	27 (29.3%)	33 (11.3%)
Years of experience, median (IQR)	12.0 (5.0–21.0)	10.0 (6.1–17.8)	17.0 (4.3–28.3)	28.5 (9.8–34.8)	17.0 (11.3–26.5)	12.0 (6.0–21.0)	13.0 (6.0–23.0)
**Working in a hospital with BFHI certification, *n* (%)**							
Yes	32 (36.0%)	0 (0.0%)	1 (5.0%)	1 (5.6%)	13 (72.2%)	15 (16.3%)	62 (21.3%)
No	18 (20.2%)	2 (3.7%)	2 (10.0%)	0 (0.0%)	1 (5.6%)	39 (42.4%)	62 (21.3%)
Do not know	39 (43.8%)	0 (0.0%)	1 (5.0%)	3 (16.7%)	4 (22.2%)	1 (1.1%)	48 (16.5%)
Not working in a hospital	0 (0.0%)	52 (96.3%)	16 (80.0%)	14 (77.8%)	0 (0.0%)	37 (40.2%)	119 (40.9%)
**Estimated personal knowledge on the use of medication during breastfeeding, *n* (%)**							
Very poor	0 (0.0%)	0 (0.0%)	1 (5.0%)	1 (5.6%)	0 (0.0%)	0 (0.0%)	2 (0.7%)
Poor	8 (9.0%)	2 (3.7%)	2 (10.0%)	5 (27.8%)	3 (16.7%)	6 (6.5%)	26 (8.9%)
Mediocre	41 (46.1%)	18 (33.3%)	9 (45.0%)	10 (55.6%)	6 (33.3%)	30 (32.6%)	114 (39.2%)
Good	36 (40.4%)	29 (53.7%)	5 (25.0%)	10 (11.1%)	9 (50.0%)	50 (54.3%)	131 (45.0%)
Excellent	4 (4.5%)	5 (9.3%)	3 (15.0%)	0 (0.0%)	0 (0.0%)	6 (6.5%)	18 (6.2%)

* Calculated based on the number of participants having children. **SD**: standard deviation; **FF**: formula fed; **BF:** breastfed; **NA**: not assessed; **LC**: lactation consultant; **IBCLC**: International Board-Certified Lactation Consultant; **IQR**: interquartile range; **BFHI**: Baby-Friendly Hospital Initiative.

**Table 2:** Beliefs about breastfeeding and its health benefits. Data shown as number (%).

	Medical Specialists (*n* = 89)	Pharmacists (*n* = 54)	General Practitioners (*n* = 20)	Dentists (*n* = 18)	Peri-Operative Nurses (*n* = 18)	Midwives (*n* = 92)	All (*n* = 291)
**Attitude towards the WHO’s advice to breastfeed exclusively for 6 months and up to 2 years of age with appropriate nutritional supplementation**							
Very negative	0 (0.0%)	0 (0.0%)	0 (0.0%)	0 (0.0%)	0 (0.0%)	0 (0.0%)	0 (0.0%)
Negative	16 (18.0%)	2 (3.7%)	0 (0.0%)	0 (0.0%)	2 (11.1%)	3 (3.3%)	23 (7.9%)
Neutral	34 (38.2%)	14 (25.9%)	4 (20.0%)	10 (55.6%)	4 (22.2%)	5 (5.4%)	71 (24.4%)
Positive	31 (34.8%)	23 (42.6%)	9 (45.0%)	6 (33.3%	4 (22.2%)	38 (41.3%)	111 (38.1%)
Very positive	8 (9.0%)	15 (27.8%)	7 (35.0%)	2 (11.1%)	8 (44.4%)	46 (50.0%)	86 (29.6%)
**Agreement with the following statements about breastfeeding**							
BF provides health advantages for both mother and child and is superior to FF	82 (92.1%)	52 (96.3%)	19 (95.0%)	15 (83.3%)	17 (94.4%)	91 (98.9%)	276 (94.8%)
BF is equivalent to FF for both mother and child	6 (6.7%)	2 (3.7%)	1 (5.0%)	3 (16.7%)	1 (5.6%)	0 (0.0%)	13 (4.5%)
BF provides health disadvantages for both mother and child and is inferior to FF	1 (1.1%)	0 (0.0%)	0 (0.0%)	0 (0.0%)	0 (0.0%)	1 (1.1%)	2 (0.7%)
**Perceived role as a health care provider towards breastfeeding**							
Encouraging BF and its continuation	43 (48.3%)	20 (37.0%)	14 (70.0%)	5 (27.8%)	14 (77.8%)	77 (83.7%)	173 (59.5%)
Encouraging BF and its continuation, but only when the patient asks for advice	41 (46.1%)	34 (63.0%)	6 (30.0%)	7 (38.9%)	3 (16.7%)	15 (16.3%)	106 (36.4%)
HCPs do not play an important role in this topic	5 (5.6%)	0 (0.0%)	0 (0.0%)	6 (33.3%)	1 (5.6%)	0 (0.0%)	12 (4.1%)
**Considered importance of continuation of breastfeeding after a surgical procedure**							
Not important at all	0 (0.0%)	0 (0.0%)	0 (0.0%)	0 (0.0%)	0 (0.0%)	0 (0.0%)	0 (0.0%)
Not important	0 (0.0%)	1 (1.9%)	0 (0.0%)	0 (0.0%)	0 (0.0%)	0 (0.0%)	1 (0.3%)
Neutral	5 (5.6%)	7 (13.0%)	5 (25.0%)	6 (33.3%)	0 (0.0%)	0 (0.0%)	23 (7.9%)
Important	54 (60.7%)	30 (55.6%)	8 (40.0%)	8 (44.4%)	6 (33.3%)	26 (28.3%)	132 (54.4%)
Very important	30 (33.7%)	16 (29.6%)	7 (35.0%)	4 (22.2%)	12 (66.7%)	66 (71.7%)	135 (46.4%)

**WHO**: World Health Organization; **BF**: breastfeeding; **FF**: formula feeding; **HCP**: health care provider.

**Table 3:** Knowledge of health care providers (HCPs) on guidelines concerning breastfeeding women undergoing (surgical) procedures and sources to evaluate medication compatibility with breastfeeding. Data shown as number (%).

	Medical Specialists (*n* = 89)	Pharmacists (*n* = 54)	General Practitioners (*n* = 20)	Dentists (*n* = 18)	Peri-Operative Nurses (*n* = 18)	Midwives (*n* = 92)	All (*n* = 291)
**Perception about availability of simple and clear protocols in general for HCPS considering breastfeeding women undergoing a (surgical) procedure**							
Yes, simple and clear protocols are available	41 (46.1%)	6 (11.1%)	2 (10.0%)	3 (16.7%)	2 (11.1%)	17 (18.5%)	71 (24.4%)
No, simple and clear protocols are not available	48 (53.9%)	48 (88.9%)	18 (90.0%)	15 (83.3%)	16 (88.9%)	75 (81%)	220 (75.6%)
**Availability of a protocol at the hospital where the HCP works considering breastfeeding women undergoing a (surgical) procedure**							
Yes, a protocol is available	37 (41.6%)	NA ^‡^	1 (5.0%)	NA **	2 (11.1%)	14 (15.2%)	54 (24.7%) *
No, there is no protocol available	21 (23.6%)	NA	0 (0.0%)	NA	10 (55.6%)	30 (32.6%)	61 (27.9%) *
I do not know whether a protocol is available	31 (34.8%)	NA	3 (15.0%)	NA	6 (33.3%)	12 (13.0%)	52 (23.7%) *
I do not work at a hospital	0 (0.0%)	NA	16 (80.0%)	NA	0 (0.0%)	36 (39.1%)	52 (23.7%) *
**Familiarity with existing guidelines or protocols considering breastfeeding women undergoing a (surgical) procedure (multiple answers possible)**							
Breastfeeding guidelines from the World Health Organization [13]	23 (25.8%)	13 (24.1%)	2 (10.0%)	4 (20.0%)	7 (38.9%)	45 (48.9%)	94 (32.3%)
Breastfeeding guidelines from the Nederlands Huisartsen Genootschap [14]	11 (12.4%)	13 (24.1%)	12 (60.0%)	1 (5.0%)	2 (11.1%)	6 (6.5%)	45 (15.5%)
Guidelines from the American Society of Anesthesiologists [15]	31 (34.8%)	0 (0.0%)	0 (0.0%)	2 (10.0%)	0 (0.0%)	3 (3.3%)	36 (12.4%)
Guidelines from the Royal College of Obstetricians and Gynecologists [16]	22 (24.7%)	0 (0.0%)	0 (0.0%)	0 (0.0%)	3 (16.7%)	4 (4.3%)	29 (10.0%)
Breastfeeding guidelines from the American Academy of Pediatrics [17]	7 (7.9%)	1 (1.9%)	0 (0.0%)	0 (0.0%)	2 (11.1%)	14 (15.2%)	24 (8.2%)
Guideline on Anesthesia and Sedation in Breastfeeding Women 2020 [18]	13 (14.6%)	0 (0.0%)	0 (0.0%)	0 (0.0%)	2 (11.1%)	9 (9.8%)	24 (8.2%)
Breastfeeding guidelines from the Centers of Disease Control [19]	7 (7.9%)	1 (1.9%)	2 (10.0%)	0 (0.0%)	1 (5.6%)	6 (6.5%)	17 (5.8%)
Antenatal and postnatal analgesia [20]	5 (5.6%)	0 (0.0%)	0 (0.0%)	0 (0.0%)	2 (11.1%)	1 (1.1%)	8 (2.7%)
Other	15 (16.9%)	11 (20.4%)	5 (25.0%)	3 (15.0%)	1 (5.6%)	10 (10.9%)	45 (15.5%)
I am not familiar with any guideline	8 (9.0%)	0 (0.0%)	0 (0.0%)	8 (40.0%)	5 (27.8%)	33 (25.9%)	54 (18.6%)
**Sources used to determine the safety of medication in (surgical) procedures for a breastfeeding woman (multiple answers possible)**							
Cybele [21]	33 (37.1%)	43 (79.6%)	13 (65%)	0 (0.0%)	3 (16.7%)	46 (50.0%)	138 (47.4%)
Lareb [22]	24 (27.0%)	37 (68.5%)	3 (15.0%)	0 (0.0%)	3 (16.7%)	28 (30.4%)	95 (32.6%)
Summary of Product Characteristics	20 (22.5%)	25 (46.3%)	6 (30.0%)	8 (44.4%)	4 (22.2%)	16 (17.4%)	79 (21.1%)
Lactmed [23]	21 (23.6%)	11 (20.4%)	0 (0.0%)	0 (0.0%)	0 (0.0%)	41 (44.6%)	73 (25.1%)
Belgian Commented Drug Repertory [24]	52 (58.4%)	25 (46.3%)	12 (60.0%)	13 (72.2%)	6 (33.3%)	0 (0.0%)	18 (37.1%)
Commentary Pharmcovigilance [25]	0 (0.0%)	11 (20.4%)	5 (25.0%)	0 (0.0%)	1 (5.6%)	1 (1.1%)	18 (6.2%)
E-Lactancia [26]	5 (5.6%)	8 (14.8%)	2 (10.0%)	0 (0.0%)	0 (0.0%)	0 (0.0%)	15 (5.2%)
InfantRisk Center [27]	1 (1.1%)	1 (1.9%)	0 (0.0%)	0 (0.0%)	0 (0.0%)	7 (7.6%)	9 (3.1%)
Motherisk [28]	1 (1.1%)	6 (11.1%)	0 (0.0%)	0 (0.0%)	0 (0.0%)	1 (1.1%)	8 (2.7%)
British National Formulary (BNF) [29,30]	1 (1.1%)	0 (0.0%)	0 (0.0%)	0 (0.0%)	0 (0.0%)	1 (1.1%)	2 (0.7%)
Medicines’ Information Leaflet	0 (0.0%)	0 (0.0%)	0 (0.0%)	0 (0.0%)	0 (0.0%)	0 (0.0%)	0 (0.0%)
Other, including local hospital protocol and scientific publications	21 (23.6%)	10 (18.5%)	9 (45.0%)	4 (22.2%)	7 (38.9%)	40 (43.5%)	91 (31.3%)

* Calculated on 219 participants; ^‡^ not applicable: only community pharmacists were included. ** Dentists generally work in private settings and were therefore not questioned about this item; **NA**: not assessed.

**Table 4:** Current advice on peri-surgical measures for breastfeeding women. Data shown as number (%).

	Medical Specialists (*n* = 89)	Pharmacists (*n* = 54)	General Practitioners (*n* = 20)	Dentists (*n* = 18)	Peri-Operative Nurses (*n* = 18)	Midwives (*n* = 92)	All (*n* = 291)
**Frequency with which the HCP inquires with a woman of fertile age about breastfeeding**							
0–20%	40 (44.9%)	36 (66.7%)	9 (45.0%)	11 (61.1%)	6 (33.3%)	NA ^‡^	102 (51.3%) *
20–40%	13 (14.6%)	4 (7.4%)	4 (20.0%)	3 (16.7%)	1 (5.6%)	NA	25 (12.6%) *
40–60%	8 (9.0%)	6 (11.1%)	0 (0.0%)	2 (11.1%)	1 (5.6%)	NA	17 (8.5%) *
60–80%	11 (12.4%)	6 (11.1%)	4 (20.0%)	0 (0.0%)	1 (5.6%)	NA	22 (11.1%) *
80–100%	17 (19.1%)	2 (3.7%)	3 (15.0%)	2 (11.1%)	9 (50.0%)	NA	33 (16.6%) *
**Advice given to a breastfeeding woman undergoing a (surgical) procedure**							
I always give the same advice	21 (23.6%)	2 (3.7%)	2 (10.0%)	NA	1 (5.6%)	1 (1.1%)	27 (9.9%) ^†^
I try to obtain more information before giving advice	49 (23.6%)	41 (75.9%)	10 (50.0%)	NA	11 (61.1%)	81 (88.0%)	192 (70.3%) ^†^
I never give advice on this topic	7 (7.9%)	7 (13.0%)	5 (25.0%)	NA	3 (16.7%)	0 (0.0%)	22 (8.1%) ^†^
Other	12 (13.5%)	4 (7.4%)	3 (15.0%)	NA	3 (16.7%)	10 (10.9%)	32 (11.7%) ^†^
**Which of the following peri-surgical measures does/do the HCP advise to a breastfeeding woman undergoing a (surgical) procedure (multiple answers possible)**							
Bringing a responsible caretaker for the nursing child	46 (51.7%)	4 (7.4%)	2 (10.0%)	NA	7 (38.9%)	23 (25.0%)	82 (30.0%) ^†^
Being planned in as the first patient of the day	11 (12.4%)	2 (3.7%)	3 (15.0%)	NA	3 (16.7%)	17 (18.5%)	36 (13.2%) ^†^
Express milk before the procedure and store for the day of the procedure	21 (23.6%)	26 (48.1%)	8 (40.0%)	NA	9 (50.0%)	59 (64.1%)	123 (45.1%) ^†^
Breastfeed or express milk right before the procedure	22 (24.7%)	20 (37.0%)	8 (40.0%)	NA	9 (50.0%)	71 (77.2%)	129 (47.3%) ^†^
Breastfeed or express milk as soon as awake and alert	22 (24.7%)	6 (11.1%)	2 (10.0%)	NA	4 (22.2%)	52 (56.5%)	87 (31.9%) ^†^
Express milk during the postoperative break in the same scheme as the infant would drink	6 (6.7%)	21 (38.9%)	6 (30.0%)	NA	7 (38.9%)	40 (43.5%)	80 (29.3%) ^†^
I do not give any advice on this topic	17 (19.1%)	19 (35.2%)	3 (15.0%)	NA	4 (22.2%)	6 (6.5%)	54 (19.8%) ^†^

* Calculated on 199 participants. ^†^ Calculated on 273 participants. ^‡^ The midwife is aware of the breastfeeding status of the woman she supervises. **HCP**: health care provider; **NA**: not assessed.

## Data Availability

The data presented in this study are available on request from the corresponding author. The data are not publicly available due to specifications in the approval of the ethical committee.

## Supplementary Materials

The following supporting information can be downloaded at: https://www.mdpi.com/article/10.3390/ijerph20043379/s1, Table S1: General questions on medication use and prescribing/dispensing practices for breastfeeding women. Table S2: Medication-specific knowledge about compatibility of analgesics used during (surgical) procedures in breastfeeding women; Table S3: Medication-specific knowledge about compatibility of adjuvants used during (surgical) procedures in breastfeeding women; Table S4: Medication-specific knowledge about compatibility of antiaggregants and anticoagulants used during (surgical) procedures in breastfeeding women; Table S5: Medication-specific knowledge about compatibility of antibiotics used during (surgical) procedures in breastfeeding women; Table S6: Medication-specific knowledge about compatibility of (local) anesthetics used during (surgical) procedures in breastfeeding women; Table S7: Medication-specific knowledge about compatibility of antiemetics used during (surgical) procedures in breastfeeding women.
