# Supplementary material for: Health Care Providers’ Perspective and Knowledge about Peri-Surgical Medication and Practices in Breastfeeding Women
Source: Int J Environ Res Public Health. 2023 Feb 15;20(4):3379. doi: 10.3390/ijerph20043379 (PMC9964632; doi:10.3390/ijerph20043379)
Supplement: Supplementary file 1 [file ijerph-20-03379-s001.zip › ijerph-2180272-supplementary.pdf]

Supplementary Material

Table S1: General questions on medication use and prescribing/dispensing practices for breastfeeding women. Data shown as number (%).

	Medical specialists (n=89)	Pharmacists (n=54)	General practitioners (n=20)	Dentists (n=18)	Perioperative nurses (n=18)	Midwives (n=92)	All (n=291)
**To what extent are you inclined to prescribe medication for a breastfeeding woman undergoing a (surgical) procedure?**							
Very inclined	9 (10.1%)	NA†	0 (0.0%)	0 (0.0%)	0 (0.0%)	1 (1.1%)	10 (4.2%)*
Inclined	28 (31.5%)	NA	2 (10.0%)	4 (22.2%)	2 (11.1%)	13 (14.1%)	49 (20.7%)*
Neutral	42 (47.2%)	NA	9 (45.0%)	4 (22.2%)	1 (5.6%)	30 (32.6%)	86 (36.3%)*
Not inclined	8 (9.0%)	NA	5 (20.0%)	9 (50.0%)	4 (22.2%)	17 (18.5%)	43 (18.1%)*
Not inclined at all	1 (1.1%)	NA	0 (0.0%)	1 (5.6%)	3 (16.7%)	2 (2.2%)	7 (3.0%)*
Not applicable	1 (1.1%)	NA	4 (20.0%)	0 (0.0%)	8 (44.4%)	29 (32%)	42 (40.5%)*
**Use of the following PK factors to determine the safety of specific medication for a breastfeeding woman when lacking sufficient information about the medication (Multiple answers were**							
I never give advice on this topic	37 (41.6%)	22 (40.7%)	14 (70.0%)	14 (77.8%)	12 (66.7%)	35 (38.0%)	134 (46.0%)
The half-life ($T_{1/2}$)	37 (41.6%)	29 (53.7%)	4 (20.0%)	3 (16.7%)	5 (27.8%)	51 (55.4%)	129 (44.3%)
The infant's age	20 (22.5%)	17 (31.5%)	1 (5.0%)	0 (0.0%)	1 (5.6%)	32 (34.8%)	71 (24.4%)
The milk-plasma AUC ratio (M/P)	21 (23.6%)	18 (33.3%)	1 (5.0%)	0 (0.0%)	4 (22.2%)	21 (22.8%)	65 (22.3%)
The relative infant dose (RID)	7 (7.9%)	11 (20.4%)	1 (5.0%)	0 (0.0%)	2 (11.1%)	20 (21.7%)	41 (14.1%)
The molecular weight (MW)	6 (6.7%)	7 (13.0%)	1 (5.0%)	1 (5.6%)	0 (0.0%)	17 (18.5%)	32 (11.0%)
The absolute infant dose (AID)	4 (4.5%)	7 (13.0%)	1 (5.0%)	0 (0.0%)	0 (0.0%)	15 (16.3%)	27 (9.3%)
The volume of distribution (Vd)	5 (5.6%)	4 (7.4%)	0 (0.0%)	0 (0.0%)	1 (5.6%)	5 (5.4%)	15 (5.2%)
The pKa	6 (6.7%)	1 (1.9%)	0 (0.0%)	0 (0.0%)	0 (0.0%)	8 (8.7%)	15 (5.2%)
**Frequency of consulting other HCPs when giving advice on the use of medication during a (surgical) procedure for a breastfeeding woman**							
Always	4 (4.5%)	6 (11.1%)	2 (10.0%)	1 (5.6%)	5 (27.8%)	18 (19.6%)	36 (12.4%)
Often	14 (15.7%)	5 (9.3%)	2 (10.0%)	1 (5.6%)	5 (27.8)	22 (23.9%)	49 (16.8%)
Regularly	20 (22.5%)	8 (14.8%)	2 (10.0%)	3 (16.7%)	3 (16.7%)	23 (25.0%)	59 (20.3%)
Sometimes	44 (49.4%)	18 (33.3%)	7 (35.0%)	10 (55.6%)	4 (22.2%)	27 (29.3%)	110 (37.8%)
Never	7 (7.9%)	17 (31.5%)	7 (35.0%)	3 (16.7%)	1 (5.6%)	2 (2.2%)	37 (12.7%)

**Calculated on 273 participants. †In Belgium, pharmacists are not allowed to prescribe medication. Therefore this question was omitted in the questionnaire for pharmacists.*

***HCP**: Health Care Provider; **PK**: pharmacokinetic.*

Table S2: Medication-specific knowledge about compatibility of analgesics used during (surgical) procedures in breastfeeding women. Correct answer (when clear advice) is underlined. Data shown as number (%).

		I never dispense/work with this medicine	This medicine is contra-indicated, so I advise to cessate breastfeeding	This medicine is contra-indicated, so I dispense/work with another medicine	This medicine is compatible	I do not know, I need to look it up
**Analgesics**						
**Paracetamol**	**SP (89)**	0 (0.0%)	0 (0.0%)	0 (0.0%)	89 (100.0%)	0 (0.0%)
	**Pharmacists (54)**	1 (1.9%)	0 (0.0%)	0 (0.0%)	53 (98.1%)	0 (0.0%)
	**GPs (20)**	0 (0.0%)	0 (0.0%)	0 (0.0%)	20 (100.0%)	0 (0.0%)
	**Dentists (18)**	4 (22.2%)	0 (0.0%)	0 (0.0%)	14 (77.8%)	0 (0.0%)
	**Nurses (18)**	0 (0.0%)	0 (0.0%)	0 (0.0%)	16 (88.9%)	2 (11.1%)
	**Midwifes (92)**	0 (0.0%)	0 (0.0%)	0 (0.0%)	92 (100.0%)	0 (0.0%)
	**All (291)**	**5 (1.7%)**	**0 (0.0%)**	**0 (0.0%)**	**284 (97.6%)**	**5 (0.7%)**
**Diclofenac**	**SP (89)**	13 (14.6%)	0 (0.0%)	4 (4.5%)	68 (76.4%)	4 (4.5%)
	**Pharmacists (54)**	7 (13.0%)	1 (1.9%)	15 (27.8%)	29 (53.7%)	2 (3.7%)
	**GPs (20)**	6 (30.0%)	0 (0.0%)	8 (40.0%)	6 (30.0%)	0 (0.0%)
	**Dentists (18)**	9 (50.0%)	0 (0.0%)	3 (16.7%)	2 (11.1%)	4 (22.2%)
	**Nurses (18)**	2 (11.1%)	0 (0.0%)	4 (22.2%)	11 (61.1%)	1 (5.6%)
	**Midwifes (92)**	15 (16.3%)	0 (0.0%)	11 (12.0%)	56 (60.9%)	10 (10.9%)
	**All (291)**	**52 (17.9%)**	**1 (0.3%)**	**45 (15.5%)**	**172 (59.1%)**	**21 (7.2%)**
**Ibuprofen**	**SP (89)**	7 (7.9%)	0 (0.0%)	3 (3.4%)	75 (84.3%)	4 (4.5%
	**Pharmacists (54)**	5 (9.3%)	2 (3.7%)	4 (7.4%)	42 (77.8%)	1 (1.9%)
	**GPs (20)**	4 (20.0%)	0 (0.0%)	3 (15.0%)	13 (65.0%)	0 (0.0%)
	**Dentists (18)**	6 (33.3%)	0 (0.0%)	3 (16.7%)	7 (38.9%)	2 (11.1%)
	**Nurses (18)**	2 (11.1%)	0 (0.0%)	2 (11.1%)	13 (72.2%)	1 (5.6%)
	**Midwifes (92)**	1 (1.1%)	0 (0.0%)	5 (5.4%)	85 (92.4%)	1 (1.1%)
	**All (291)**	**25 (8.6%)**	**2 (0.7%)**	**20 (6.9%)**	**235 (80.8%)**	**9 (3.1%)**
**Buprenorphine**	**SP (89)**	25 (28.1%)	5 (5.6%)	12 (13.5%)	6 (6.7%)	41 (46.1%)
	**Pharmacists (54)**	27 (50.0%)	11 (20.4%)	8 (14.8%)	6 (11.1%)	2 (3.7%)
	**GPs (20)**	7 (35.0%)	1 (5.0%)	9 (45.0%)	1 (5.0%)	2 (10.0%)
	**Dentists (18)**	NA	NA	NA	NA	NA
	**Nurses (18)**	10 (55.6%)	0 (0.0%)	3 (16.7%)	0 (0.0%)	5 (27.8%)
	**Midwifes (92)**	NA	NA	NA	NA	NA
	**All (163)**	**69 (42.3%)**	**17 (10.4%)**	**32 (19.6%)**	**13 (8.0%)**	**50 (30.7%)**
**Fentanyl**	**SP (89)**	18 (20.2%)	14 (15.7%)	15 (16.9%)	14 (15.7%)	28 (31.5%)
	**Pharmacists (54)**	24 (44.4%)	13 (24.1%)	9 (16.7%)	5 (9.3%)	3 (5.6%)
	**GPs (20)**	8 (40.0%)	1 (5.0%)	8 (40.0%)	2 (10.0%)	1 (5.0%)
	**Dentists (18)**	NA	NA	NA	NA	NA
	**Nurses (18)**	8 (44.4%)	0 (0.0%)	5 (27.8%)	0 (0.0%)	5 (27.8%)
	**Midwifes (92)**	NA	NA	NA	NA	NA
	**All (163)**	**58 (35.6%)**	**28 (17.2%)**	**37 (22.7%)**	**21 (12.9%)**	**37 (22.7%)**
**Sufentanil**	**SP (89)**	17 (19.1%)	16 (18.0%)	4 (4.5%)	37 (41.6%)	15 (16.9%)
	**Pharmacists (54)**	28 (51.9%)	10 (18.5%)	3 (5.6%)	0 (0.0%)	13 (24.1%)
	**GPs (20)**	7 (35.0%)	0 (0.0%)	2 (10.0%)	2 (10.0%)	9 (45.0%)
	**Dentists (18)**	NA	NA	NA	NA	NA
	**Nurses (18)**	7 (38.9%)	0 (0.0%)	3 (16.7%)	4 (22.2%)	4 (22.2%)
	**Midwifes (92)**	NA	NA	NA	NA	NA
	**All (163)**	**59 (36.2%)**	**26 (16.0%)**	**12 (7.4%)**	**43 (26.4%)**	**41 (25.2%)**
**Morphine**	**SP (89)**	21 (23.6%)	11 (12.4%)	18 (20.2%)	12 (13.5%)	27 (30.3%)
	**Pharmacists (54)**	21 (38.9%)	17 (31.5%)	10 (18.5%)	2 (3.7%)	4 (7.4%)
	**GPs (20)**	10 (50.0%)	1 (5.0%)	7 (35.0%)	0 (0.0%)	2 (10.0%)
	**Dentists (18)**	NA	NA	NA	NA	NA
	**Nurses (18)**	4 (22.2%)	1 (5.6%)	7 (38.9%)	2 (11.1%)	4 (22.2%)
	**Midwifes (92)**	NA	NA	NA	NA	NA
	**All (163)**	**56 (34.4%)**	**30 (18.4%)**	**42 (25.8%)**	**16 (9.8%)**	**37 (22.7%)**
**Codeïne**	**SP (89)**	15 (16.9%)	8 (9.0%)	28 (31.5%)	6 (6.7%)	32 (36.0%)
	**Pharmacists (54)**	15 (27.8%)	13 (24.1%)	22 (40.7%)	1 (1.9%)	3 (5.6%)
	**GPs (20)**	7 (35.0%)	2 (10.0%)	8 (40.0%)	0 (0.0%)	3 (15.0%)
	**Dentists (18)**	6 (33.3%)	0 (0.0%)	7 (38.9%)	0 (0.0%)	5 (27.8%)

	**Nurses (18)**	5 (27.8%)	0 (0.0%)	8 (44.4%)	0 (0.0%)	5 (27.8%)
	**Midwifes (92)**	17 (18.5%)	0 (0.0%)	51 (55.4%)	2 (2.2%)	22 (23.9%)
	**All (291)**	**65 (22.3%)**	**23 (7.9%)**	**124 (42.6%)**	**9 (3.1%)**	**70 (24.1%)**
**Tramadol**	**SP (89)**	15 (16.9%)	9 (10.1%)	9 (10.1%)	46 (51.7%)	10 (11.2%)
	**Pharmacists (54)**	20 (37.0%)	12 (22.2%)	12 (22.2%)	6 (11.1%)	4 (7.4%)
	**GPs (20)**	9 (45.0%)	1 (5.0%)	8 (40.0%)	2 (10.0%)	0 (0.0%)
	**Dentists (18)**	5 (27.8%)	0 (0.0%)	6 (33.3%)	1 (5.6%)	6 (33.3%)
	**Nurses (18)**	5 (27.8%)	0 (0.0%)	5 (27.8%)	5 (27.8%)	3 (16.7%)
	**Midwifes (92)**	23 (25.0%)	0 (0.0%)	27 (29.3%)	23 (25.0%)	19 (20.7%)
	**All (291)**	**77 (26.5%)**	**22 (7.6%)**	**67 (23.0%)**	**83 (28.5%)**	**42 (14.4%)**
**Oxycodone**	**SP (89)**	16 (18.0%)	10 (11.2%)	28 (31.5%)	7 (7.9%)	28 (31.5%)
	**Pharmacists (54)**	23 (42.6%)	15 (27.8%)	11 (20.4%)	1 (1.9%)	4 (7.4%)
	**GPs (20)**	8 (40.0%)	1 (5.0%)	9 (45.0%)	0 (0.0%)	2 (10.0%)
	**Dentists (18)**	NA	NA	NA	NA	NA
	**Nurses (18)**	7 (38.9%)	0 (0.0%)	6 (33.3%)	0 (0.0%)	5 (27.8%)
	**Midwifes (92)**	NA	NA	NA	NA	NA
	**All (181)**	**54 (29.8%)**	**26 (14.4%)**	**54 (29.8%)**	**8 (4.4%)**	**39 (21.5%)**
**Piritramide**	**SP (89)**	17 (19.1%)	15 (16.9%)	15 (16.9%)	30 (33.7%)	12 (13.5%)
	**Pharmacists (54)**	26 (48.1%)	10 (18.5%)	7 (13.0%)	1 (1.9%)	10 (18.5%)
	**GPs (20)**	7 (35.0%)	1 (5.0%)	5 (25.0%)	1 (5.0%)	6 (30.0%)
	**Dentists (18)**	NA	NA	NA	NA	NA
	**Nurses (18)**	4 (22.2%)	1 (5.6%)	8 (44.4%)	1 (5.6%)	4 (22.2%)
	**Midwifes (92)**	NA	NA	NA	NA	NA
	**All (181)**	**54 (29.8%)**	**27 (14.9%)**	**35 (19.3%)**	**33 (18.2%)**	**32 (17.7%)**

***GP**: General Practitioner; **NA**: Not Assessed; **SP**: Specialty Physician*

Table S3: Medication-specific knowledge about compatibility of adjuvants used during (surgical) procedures in breastfeeding women. Correct answer (when clear advice) is underlined. Data shown as number (%).

		I never dispense/work with this medicine	This medicine is contra-indicated, so I advise to cessate breastfeeding	This medicine is contra-indicated, so I dispense/work with another medicine	This medicine is compatible	I do not know, I need to look it up
**Adjuvants**						
**Atracurium**	**SP (89)**	15 (16.9%)	2 (2.2%)	0 (0.0%)	38 (42.7%)	34 (38.2%)
	**Pharmacists (54)**	28 (51.9%)	2 (3.7%)	0 (0.0%)	2 (3.7%)	22 (40.7%)
	**GPs (20)**	NA	NA	NA	NA	NA
	**Dentists (18)**	NA	NA	NA	NA	NA
	**Nurses (18)**	10 (55.6%)	0 (0.0%)	0 (0.0%)	1 (5.6%)	7 (38.9%)
	**Midwifes (92)**	NA	NA	NA	NA	NA
	**All (161)**	**53 (32.9%)**	**4 (2.5%)**	**0 (0.0%)**	**41 (25.5%)**	**63 (39.1%)**
**Rocuronium**	**SP (89)**	14 (15.7%)	3 (3.4%)	0 (0.0%)	54 (60.7%)	18 (20.2%)
	**Pharmacists (54)**	28 (51.9%)	1 (1.9%)	0 (0.0%)	2 (3.7%)	23 (42.6%)
	**GPs (20)**	NA	NA	NA	NA	NA
	**Dentists (18)**	NA	NA	NA	NA	NA
	**Nurses (18)**	7 (38.9%)	0 (0.0%)	1 (5.6%)	3 (16.7%)	7 (38.9%)
	**Midwifes (92)**	NA	NA	NA	NA	NA
	**All (161)**	**49 (30.4%)**	**4 (2.5%)**	**1 (0.6%)**	**59 (36.6%)**	**48 (29.8%)**
**Cisatracurium**	**SP (89)**	14 (15.7%)	2 (2.2%)	1 (1.1%)	48 (53.9%)	24 (27.0%)
	**Pharmacists (54)**	28 (51.9%)	2 (3.7%)	0 (0.0%)	2 (3.7%)	22 (40.7%)
	**GPs (20)**	NA	NA	NA	NA	NA
	**Dentists (18)**	NA	NA	NA	NA	NA
	**Nurses (18)**	8 (44.4%)	1 (5.6%)	1 (5.6%)	2 (11.1%)	6 (33.3%)
	**Midwifes (92)**	NA	NA	NA	NA	NA
	**All (161)**	**50 (31.1%)**	**5 (3.1%)**	**2 (1.2%)**	**52 (32.3%)**	**52 (32.3%)**
**Clonidine**	**SP (89)**	34 (38.2%)	2 (2.2%)	8 (9.0%)	18 (20.2%)	27 (30.3%)
	**Pharmacists (54)**	37 (68.5%)	4 (7.4%)	6 (11.1%)	1 (1.9%)	6 (11.1%)
	**GPs (20)**	NA	NA	NA	NA	NA
	**Dentists (18)**	NA	NA	NA	NA	NA
	**Nurses (18)**	9 (50.0%)	0 (0.0%)	0 (0.0%)	2 (11.1%)	7 (38.9%)
	**Midwifes (92)**	NA	NA	NA	NA	NA
	**All (161)**	**80 (49.7%)**	**6 (3.7%)**	**14 (8.7%)**	**21 (13.0%)**	**40 (24.8%)**
**Propofol**	**SP (89)**	12 (13.5%)	5 (5.6%)	0 (0.0%)	55 (61.8%)	17 (19.1%)
	**Pharmacists (54)**	26 (48.1%)	3 (5.6%)	1 (1.9%)	2 (3.7%)	22 (40.7%)
	**GPs (20)**	NA	NA	NA	NA	NA
	**Dentists (18)**	NA	NA	NA	NA	NA
	**Nurses (18)**	8 (44.4%)	0 (0.0%)	1 (5.6%)	2 (11.1%)	7 (38.9%)
	**Midwifes (92)**	NA	NA	NA	NA	NA
	**All (161)**	**46 (28.6%)**	**8 (5.0%)**	**2 (1.2%)**	**59 (36.6%)**	**46 (28.6%)**
**Ketamine**	**SP (89)**	29 (32.6%)	5 (5.6%)	9 (10.1%)	20 (22.5%)	26 (29.2%)
	**Pharmacists (54)**	23 (42.6%)	5 (9.3%)	5 (9.3%)	0 (0.0%)	21 (38.9%)
	**GPs (20)**	NA	NA	NA	NA	NA
	**Dentists (18)**	NA	NA	NA	NA	NA
	**Nurses (18)**	8 (44.4%)	0 (0.0%)	3 (16.7%)	1 (5.6%)	6 (33.3%)
	**Midwifes (92)**	NA	NA	NA	NA	NA
	**All (161)**	**60 (37.3%)**	**10 (6.2%)**	**17 (10.6%)**	**21 (13.0%)**	**53 (32.9%)**
**Midazolam**	**SP (89)**	22 (24.7%)	6 (6.7%)	11 (12.4%)	27 (30.3%)	23 (25.8%)
	**Pharmacists (54)**	27 (50.0%)	7 (13.0%)	3 (5.6%)	1 (1.9%)	16 (29.6%)
	**GPs (20)**	NA	NA	NA	NA	NA
	**Dentists (18)**	NA	NA	NA	NA	NA
	**Nurses (18)**	8 (44.4%)	0 (0.0%)	2 (11.1%)	1 (5.6%)	7 (38.9%)
	**Midwifes (92)**	NA	NA	NA	NA	NA
	**All (161)**	**57 (35.4%)**	**13 (8.1%)**	**16 (9.9%)**	**29 (18.0%)**	**46 (28.6%)**

***GP**: General Practitioner; **NA**: Not Assessed; **SP**: Specialty Physician*

Table S4: Medication-specific knowledge about compatibility of Antiaggregants and anticoagulants used during (surgical) procedures in breastfeeding women. Correct answer (when clear advice) is underlined. Data shown as number (%).

		I never dispense/work with this medicine	This medicine is contra-indicated, so I advise to cessate breastfeeding	This medicine is contra-indicated, so I dispense/work with another medicine	This medicine is compatible	I do not know, I need to look it up
**Antiaggregants and anticoagulants**						
**Acetylsalicylic acid as antiaggregant (30 à 100 mg per day)**	**SP (89)**	23 (25.8%)	0 (0.0%)	3 (3.4%)	55 (61.8%)	8 (9.0%)
	**Pharmacists (54)**	15 (27.8%)	2 (3.7%)	9 (16.7%)	26 (48.1%)	2 (3.7%)
	**GPs (20)**	8 (40.0%)	2 (10.0%)	2 (10.0%)	7 (35.0%)	1 (5.0%)
	**Dentists (18)**	6 (33.3%)	0 (0.0%)	0 (0.0%)	3 (16.7%)	9 (50.0%)
	**Nurses (18)**	8 (44.4%)	0 (0.0%)	2 (11.1%)	5 (27.8%)	3 (16.7%)
	**Midwifes (92)**	41 (44.6%)	0 (0.0%)	9 (9.8%)	27 (29.3%)	15 (16.3%)
	**All (291)**	**101 (34.7%)**	**4 (1.4%)**	**25 (8.6%)**	**<u>123 (42.3%)</u>**	**38 (13.1%)**
**Heparine**	**SP (89)**	38 (42.7%)	2 (2.2%)	2 (2.2%)	37 (41.6%)	10 (11.2%)
	**Pharmacists (54)**	21 (38.9%)	0 (0.0%)	2 (3.7%)	29 (53.7%)	2 (3.7%)
	**GPs (20)**	13 (65.0%)	0 (0.0%)	1 (5.0%)	4 (20.0%)	2 (10.0%)
	**Dentists (18)**	6 (33.3%)	0 (0.0%)	1 (5.6%)	0 (0.0%)	11 (61.1%)
	**Nurses (18)**	10 (55.6%)	0 (0.0%)	0 (0.0%)	5 (27.8%)	3 (16.7%)
	**Midwifes (92)**	53 (57.6%)	0 (0.0%)	3 (3.3%)	23 (25.0%)	13 (14.1%)
	**All (291)**	**141 (48.5%)**	**2 (0.7%)**	**9 (3.1%)**	**<u>98 (33.7%)</u>**	**41 (14.1%)**
**Nadroparine**	**SP (89)**	19 (21.3%)	1 (1.1%)	1 (1.1%)	63 (70.8%)	5 (5.6%)
	**Pharmacists (54)**	20 (37.0%)	0 (0.0%)	3 (5.6%)	30 (55.6%)	1 (1.9%)
	**GPs (20)**	11 (55.0%)	0 (0.0%)	1 (5.0%)	7 (35.0%)	1 (5.0%)
	**Dentists (18)**	6 (33.3%)	0 (0.0%)	0 (0.0%)	0 (0.0%)	12 (66.7%)
	**Nurses (18)**	6 (33.3%)	0 (0.0%)	0 (0.0%)	10 (55.6%)	2 (11.1%)
	**Midwifes (92)**	15 (16.3%)	0 (0.0%)	0 (0.0%)	73 (79.3%)	4 (4.3%)
	**All (291)**	**77 (26.5%)**	**1 (0.3%)**	**5 (1.7%)**	**<u>183 (62.9%)</u>**	**25 (8.6%)**
**Enoxaparine**	**SP (89)**	14 (15.7%)	1 (1.1%)	0 (0.0%)	71 (79.8%)	3 (3.4%)
	**Pharmacists (54)**	18 (33.3%)	0 (0.0%)	1 (1.9%)	34 (63.0%)	1 (1.9%)
	**GPs (20)**	10 (50.0%)	0 (0.0%)	1 (5.0%)	8 (40.0%)	1 (5.0%)
	**Dentists (18)**	6 (33.3%)	0 (0.0%)	0 (0.0%)	0 (0.0%)	12 (66.7%)
	**Nurses (18)**	6 (33.3%)	0 (0.0%)	0 (0.0%)	10 (55.6%)	2 (11.1%)
	**Midwifes (92)**	9 (9.8%)	0 (0.0%)	0 (0.0%)	80 (87.0%)	3 (3.3%)
	**All (291)**	**63 (21.6%)**	**1 (0.3%)**	**2 (0.7%)**	**<u>203 (69.8%)</u>**	**22 (7.6%)**

***GP**: General Practitioner; **NA**: Not Assessed; **SP**: Specialty Physician*

Table S5: Medication-specific knowledge about compatibility of antibiotics used during (surgical) procedures in breastfeeding women. Correct answer (when clear advice) is underlined. Data shown as number (%).

		I never dispense/work with this medicine	This medicine is contra-indicated, so I advise to cessate breastfeeding	This medicine is contra-indicated, so I dispense/work with another medicine	This medicine is compatible	I do not know, I need to look it up
**Antibiotics**						
**Amoxicillin**	**SP (89)**	15 (16.9%)	0 (0.0%)	1 (1.1%)	72 (80.9%)	1 (1.1%)
	**Pharmacists (54)**	1 (1.9%)	2 (3.7%)	0 (0.0%)	51 (94.4%)	0 (0.0%)
	**GPs (20)**	1 (5.0%)	0 (0.0%)	0 (0.0%)	19 (95.0%)	0 (0.0%)
	**Dentists (18)**	3 (16.7%)	0 (0.0%)	2 (11.1%)	13 (72.2%)	0 (0.0%)
	**Nurses (18)**	3 (16.7%)	1 (5.6%)	0 (0.0%)	13 (72.2%)	1 (5.6%)
	**Midwifes (92)**	7 (7.6%)	0 (0.0%)	0 (0.0%)	79 (85.9%)	6 (6.5%)
	**All (291)**	**30 (10.3%)**	**3 (1.0%)**	**3 (1.0%)**	**<u>247 (84.9%)</u>**	**8 (2.7%)**
**Amoxicillin + clavulanic acid**	**SP (89)**	20 (22.5%)	1 (1.1%)	6 (6.7%)	61 (68.5%)	1 (1.1%)
	**Pharmacists (54)**	8 (14.8%)	2 (3.7%)	7 (13.0%)	37 (68.5%)	0 (0.0%)
	**GPs (20)**	4 (20.0%)	1 (5.0%)	1 (5.0%)	14 (70.0%)	0 (0.0%)
	**Dentists (18)**	6 (33.3%)	0 (0.0%)	2 (11.1%)	8 (44.4%)	2 (11.1%)
	**Nurses (18)**	5 (27.8%)	1 (5.6%)	0 (0.0%)	11 (61.1%)	1 (5.6%)
	**Midwifes (92)**	9 (9.8%)	0 (0.0%)	3 (3.3%)	75 (81.5%)	5 (5.4%)
	**All (291)**	**52 (17.9%)**	**5 (1.7%)**	**19 (6.5%)**	**<u>206 (70.8%)</u>**	**9 (3.1%)**
**Cefazolin**	**SP (89)**	18 (20.2%)	1 (1.1%)	1 (1.1%)	68 (76.4%)	1 (1.1%)
	**Pharmacists (54)**	38 (70.4%)	1 (1.9%)	1 (1.9%)	8 (14.8%)	6 (11.1%)
	**GPs (20)**	11 (55.0%)	0 (0.0%)	1 (5.0%)	5 (25.0%)	3 (15.0%)
	**Dentists (18)**	NA	NA	NA	NA	NA
	**Nurses (18)**	7 (38.9%)	1 (5.6%)	0 (0.0%)	9 (50.0%)	1 (5.6%)
	**Midwifes (92)**	47 (51.1%)	0 (0.0%)	2 (2.2%)	33 (35.9%)	10 (10.9%)
	**All (273)**	**121 (44.3%)**	**3 (1.1%)**	**5 (1.8%)**	**<u>123 (45.1%)</u>**	**21 (7.7%)**
**Clarithromycin**	**SP (89)**	43 (48.3%)	2 (2.2%)	6 (6.7%)	32 (36.0%)	6 (6.7%)
	**Pharmacists (54)**	35 (64.8%)	2 (3.7%)	4 (7.4%)	13 (24.1%)	0 (0.0%)
	**GPs (20)**	11 (55.0%)	0 (0.0%)	3 (15.0%)	6 (30.0%)	0 (0.0%)
	**Dentists (18)**	8 (44.4%)	0 (0.0%)	4 (22.2%)	3 (16.7%)	3 (16.7%)
	**Nurses (18)**	11 (61.1%)	1 (5.6%)	0 (0.0%)	3 (16.7%)	3 (16.7%)
	**Midwifes (92)**	65 (70.7%)	0 (0.0%)	3 (3.3%)	13 (14.1%)	11 (12.0%)
	**All (291)**	**173 (59.5%)**	**5 (1.7%)**	**20 (6.9%)**	**<u>70 (24.1%)</u>**	**23 (7.9%)**
**Clindamycin**	**SP (89)**	39 (43.8%)	2 (2.2%)	5 (5.6%)	40 (44.9%)	3 (3.4%)
	**Pharmacists (54)**	35 (64.8%)	2 (3.7%)	5 (9.3%)	11 (20.4%)	1 (1.9%)
	**GPs (20)**	15 (75.0%)	0 (0.0%)	2 (10.0%)	3 (15.0%)	0 (0.0%)
	**Dentists (18)**	9 (50.0%)	0 (0.0%)	3 (16.7%)	5 (27.8%)	1 (5.6%)
	**Nurses (18)**	7 (38.9%)	1 (5.6%)	0 (0.0%)	8 (44.4%)	2 (11.1%)
	**Midwifes (92)**	39 (42.4%)	0 (0.0%)	2 (2.2%)	41 (44.6%)	10 (10.9%)
	**All (291)**	**144 (49.5%)**	**5 (1.7%)**	**17 (5.8%)**	**<u>108 (37.1%)</u>**	**17 (5.8%)**

GP: *General Practitioner;* ***NA***: *Not Assessed;* ***SP***: *Specialty Physician*

Table S6: Medication-specific knowledge about compatibility of (local) anesthetics used during (surgical) procedures in breastfeeding women. Correct answer (when clear advice) is underlined. Data shown as number (%).

		I never dispense/work with this medicine	This medicine is contra-indicated, so I advise to cessate breastfeeding	This medicine is contra-indicated, so I dispense/work with another medicine	This medicine is compatible	I do not know, I need to look it up
**(Local) anaesthetics**						
**Lidocaine**	**SP (89)**	7 (7.9%)	0 (0.0%)	0 (0.0%)	78 (87.6%)	4 (4.5%)
	**Pharmacists (54)**	31 (57.4%)	1 (1.9%)	1 (1.9%)	19 (35.2%)	2 (3.7%)
	**GPs (20)**	NA	NA	NA	NA	NA
	**Dentists (18)**	2 (11.1%)	1 (5.6%)	0 (0.0%)	13 (72.2%)	2 (11.1%)
	**Nurses (18)**	4 (22.2%)	0 (0.0%)	0 (0.0%)	12 (66.7%)	2 (11.1%)
	**Midwifes (92)**	32 (34.8%)	0 (0.0%)	1 (1.1%)	43 (46.7%)	16 (17.4%)
	**All (271)**	**76 (28.0%)**	**2 (0.7%)**	**2 (0.7%)**	**165 (60.9%)**	**26 (9.6%)**
**Bupivacaine**	**SP (89)**	12 (13.5%)	0 (0.0%)	0 (0.0%)	65 (73.0%)	12 (13.5%)
	**Pharmacists (54)**	32 (59.3%)	1 (1.9%)	0 (0.0%)	6 (11.1%)	15 (27.8%)
	**GPs (20)**	NA	NA	NA	NA	NA
	**Dentists (18)**	7 (38.9%)	0 (0.0%)	1 (5.6%)	2 (11.1%)	8 (44.4%)
	**Nurses (18)**	9 (50.0%)	0 (0.0%)	0 (0.0%)	5 (27.8%)	4 (22.2%)
	**Midwifes (92)**	50 (54.3%)	0 (0.0%)	1 (1.1%)	24 (26.1%)	17 (18.5%)
	**All (271)**	**110 (40.6%)**	**1 (0.4%)**	**2 (0.7%)**	**102 (37.6%)**	**56 (20.7%)**
**Levobupivacaine**	**SP (89)**	13 (14.6%)	0 (0.0%)	0 (0.0%)	59 (66.3%)	17 (19.1%)
	**Pharmacists (54)**	32 (59.3%)	1 (1.9%)	0 (0.0%)	4 (7.4%)	17 (31.5%)
	**GPs (20)**	NA	NA	NA	NA	NA
	**Dentists (18)**	NA	NA	NA	NA	NA
	**Nurses (18)**	9 (50.0%)	0 (0.0%)	0 (0.0%)	5 (27.8%)	4 (22.2%)
	**Midwifes (92)**	60 (65.2%)	0 (0.0%)	1 (1.1%)	13 (14.1%)	18 (19.6%)
	**All (271)**	**114 (42.1%)**	**1 (0.4%)**	**1 (0.4%)**	**81 (29.9%)**	**56 (20.7%)**
**Ropivacaine**	**SP (89)**	15 (16.9%)	0 (0.0%)	0 (0.0%)	62 (69.7%)	12 (13.5%)
	**Pharmacists (54)**	31 (57.4%)	1 (1.9%)	0 (0.0%)	4 (7.4%)	18 (33.3%)
	**GPs (20)**	NA	NA	NA	NA	NA
	**Dentists (18)**	7 (38.9%)	0 (0.0%)	1 (5.6%)	1 (5.6%)	9 (50.0%)
	**Nurses (18)**	10 (55.6%)	0 (0.0%)	1 (5.6%)	3 (16.7%)	4 (22.2%)
	**Midwifes (92)**	44 (47.8%)	0 (0.0%)	2 (2.2%)	29 (31.5%)	17 (18.5%)
	**All (271)**	**107 (39.5%)**	**1 (0.4%)**	**4 (1.5%)**	**99 (36.5%)**	**60 (22.1%)**
**Desflurane**	**SP (89)**	17 (19.1%)	5 (5.6%)	2 (2.2%)	40 (44.9%)	25 (28.1%)
	**Pharmacists (54)**	30 (55.6%)	3 (5.6%)	0 (0.0%)	2 (3.7%)	19 (35.2%)
	**GPs (20)**	NA	NA	NA	NA	NA
	**Dentists (18)**	NA	NA	NA	NA	NA
	**Nurses (18)**	11 (61.1%)	0 (0.0%)	0 (0.0%)	2 (11.1%)	5 (27.8%)
	**Midwifes (92)**	NA	NA	NA	NA	NA
	**All (161)**	**58 (36.0%)**	**8 (5.0%)**	**2 (1.2%)**	**44 (27.3%)**	**49 (30.4%)**
**Sevoflurane**	**SP (89)**	11 (12.4%)	6 (6.7%)	0 (0.0%)	56 (62.9%)	16 (18.0%)
	**Pharmacists (54)**	30 (55.6%)	3 (5.6%)	0 (0.0%)	2 (3.7%)	19 (35.2%)
	**GPs (20)**	NA	NA	NA	NA	NA
	**Dentists (18)**	NA	NA	NA	NA	NA
	**Nurses (18)**	10 (55.6%)	0 (0.0%)	1 (5.6%)	2 (11.1%)	5 (27.8%)
	**Midwifes (92)**	NA	NA	NA	NA	NA
	**All (161)**	**51 (31.7%)**	**9 (5.6%)**	**1 (0.6%)**	**60 (37.3%)**	**40 (24.8%)**

__GP__: General Practitioner; __NA__: Not Assessed; __SP__: Specialty Physician

Table S7: Medication-specific knowledge about compatibility of antiemetics used during (surgical) procedures in breastfeeding women. Correct answer (when clear advice) is underlined. Data shown as number (%).

		I never dispense/work with this medicine	This medicine is contra-indicated, so I advise to cessate breastfeeding	This medicine is contra-indicated, so I dispense/work with another medicine	This medicine is compatible	I do not know, I need to look it up
**Antiemetics**						
**Metoclopramide**	**SP (89)**	17 (19.1%)	1 (1.1%)	6 (6.7%)	55 (61.8%)	10 (11.2%)
	**Pharmacists (54)**	12 (22.2%)	0 (0.0%)	6 (11.1%)	36 (66.7%)	0 (0.0%)
	**GPs (20)**	7 (35.0%)	0 (0.0%)	1 (5.0%)	12 (60.0%)	0 (0.0%)
	**Dentists (18)**	NA	NA	NA	NA	NA
	**Nurses (18)**	4 (22.2%)	1 (5.6%)	0 (0.0%)	12 (66.7%)	1 (5.6%)
	**Midwifes (92)**	19 (20.7%)	0 (0.0%)	2 (2.2%)	68 (73.9%)	3 (3.3%)
	**All (273)**	**59 (21.6%)**	**2 (0.7%)**	**15 (5.5%)**	**<u>183 (67.0%)</u>**	**14 (5.1%)**
**Alizapride[1]**	**SP (89)**	23 (25.8%)	0 (0.0%)	6 (6.7%)	55 (61.8%)	5 (5.6%)
	**Pharmacists (54)**	35 (64.8%)	1 (1.9%)	12 (22.2%)	6 (11.1%)	0 (0.0%)
	**GPs (20)**	14 (70.0%)	0 (0.0%)	3 (15.0%)	2 (10.0%)	1 (5.0%)
	**Dentists (18)**	NA	NA	NA	NA	NA
	**Nurses (18)**	4 (22.2%)	1 (5.6%)	1 (5.6%)	10 (55.6%)	2 (11.1%)
	**Midwifes (92)**	29 (31.5%)	0 (0.0%)	4 (4.3%)	54 (58.7%)	5 (5.4%)
	**All (273)**	**105 (38.5%)**	**2 (0.7%)**	**26 (9.5%)**	**127 (46.5%)**	**13 (4.8%)**
**Domperidone**	**SP (89)**	33 (37.1%)	2 (2.2%)	9 (10.1%)	32 (36.0%)	13 (14.6%)
	**Pharmacists (54)**	6 (11.1%)	1 (1.9%)	11 (20.4%)	35 (64.8%)	1 (1.9%)
	**GPs (20)**	7 (35.0%)	0 (0.0%)	6 (30.0%)	7 (35.0%)	0 (0.0%)
	**Dentists (18)**	NA	NA	NA	NA	NA
	**Nurses (18)**	4 (22.2%)	1 (5.6%)	0 (0.0%)	12 (66.7%)	1 (5.6%)
	**Midwifes (92)**	10 (10.9%)	0 (0.0%)	5 (5.4%)	72 (78.3%)	5 (5.4%)
	**All (273)**	**60 (22.0%)**	**4 (1.5%)**	**31 (11.4%)**	**<u>158 (57.9%)</u>**	**20 (7.3%)**
**Ondansetron[1]**	**SP (89)**	19 (21.3%)	1 (2.2%)	6 (6.7%)	59 (66.3%)	4 (4.5%)
	**Pharmacists (54)**	35 (64.8%)	0 (0.0%)	7 (13.0%)	3 (5.6%)	9 (16.7%)
	**GPs (20)**	16 (80.0%)	0 (0.0%)	1 (5.0%)	0 (0.0%)	3 (15.0%)
	**Dentists (18)**	NA	NA	NA	NA	NA
	**Nurses (18)**	6 (33.3%)	1 (5.6%)	2 (11.1%)	6 (33.3%)	3 (16.7%)
	**Midwifes (92)**	59 (64.1%)	0 (0.0%)	4 (4.3%)	15 (16.3%)	14 (15.2%)
	**All (273)**	**135 (49.5%)**	**2 (0.7%)**	**20 (7.3%)**	**83 (30.4%)**	**33 (12.1%)**
**Droperidol[1]**	**SP (89)**	36 (40.4%)	3 (3.4%)	17 (19.1%)	16 (18.0%)	17 (19.1%)
	**Pharmacists (54)**	30 (55.6%)	2 (3.7%)	5 (9.3%)	1 (1.9%)	16 (29.6%)
	**GPs (20)**	15 (75.0%)	0 (0.0%)	1 (5.0%)	0 (0.0%)	4 (20.0%)
	**Dentists (18)**	NA	NA	NA	NA	NA
	**Nurses (18)**	11 (61.1%)	0 (0.0%)	1 (5.6%)	1 (5.6%)	5 (27.8%)
	**Midwifes (92)**	67 (72.8%)	0 (0.0%)	5 (5.4%)	3 (3.3%)	17 (18.5%)
	**All (273)**	**159 (58.2%)**	**5 (1.2%)**	**29 (10.6%)**	**21 (7.7%)**	**59 (21.6%)**

[1] The risk of administration of this drug during breastfeeding is considered possible or unknown

***GP**: General Practitioner; **NA**: Not Assessed; **SP**: Specialty Physician*
